# Identification and removal of a cryptic impurity in pomalidomide-PEG based PROTAC

**DOI:** 10.3762/bjoc.21.28

**Published:** 2025-02-18

**Authors:** Bingnan Wang, Yong Lu, Chuo Chen

**Affiliations:** 1 Department of Biochemistry, UT Southwestern Medical Center, 5323 Harry Hines Boulevard, Dallas, TX 75390-9038, USA

**Keywords:** glutarimide, IMiD, impurity, nucleophilic acyl substitution, PROTAC

## Abstract

Chemically induced dimerization is a powerful tool for studying protein function, wherein the IMiD (the “immunomodulatory drug”) class of PROTAC molecules with a PEG linker is frequently used to promote targeted protein degradation. The standard protocol for their synthesis involves nucleophilic aromatic substitution of 4-fluorothalidomide with a PEG-amine. We report herein the identification of a commonly ignored impurity generated in this process. Nucleophilic acyl substitution competes with aromatic substitution to displace glutarimide and gives a byproduct that can co-elute with the desired product on HPLC throughout the remainder of the synthesis. Scavenging with taurine is a convenient way to minimize this contamination.

## Introduction

**Figure 1:**
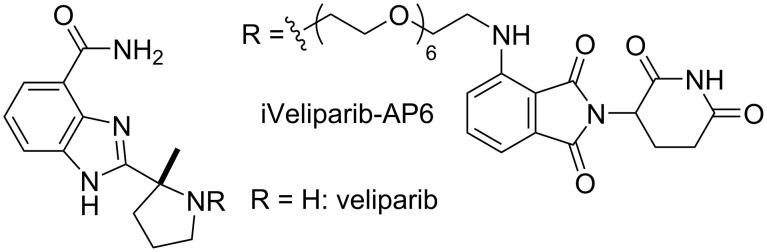
The structures of veliparib and iVeliparib-AP6.

Targeted protein degradation capitalizing on the concept of chemically induced dimerization has emerged as a new therapeutic approach recently [1]. In particular, the modularity of proteolysis targeting chimera (PROTAC) has made it a popular starting point to develop selective small-molecule degraders [2]. Currently, leveraging ubiquitination by the von Hippel–Lindau (VHL) protein or cereblon (CRBN) is the most successful method to achieve targeted protein degradation [3–4]. For initial studies, a short polyethylene glycol (PEG) linker of various lengths is typically used to build a small library to identify a lead compound. For example, iVeliparib-AP6 (Figure 1) developed through this practice is a PROTAC that degrades poly(ADP-ribose) polymerase 2 (PARP2) selectively [5].

## Results and Discussion

**Scheme 1:**
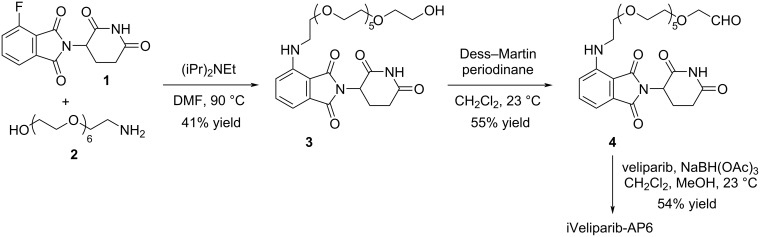
The synthetic route for iVeliparib-AP6.

The synthesis of iVeliparib-AP6 [5] starts with a nucleophilic aromatic substitution (S_N_Ar) reaction wherein 4-fluorothalidomide (**1**) reacts with amino-PEG7-OH **2** to give alcohol **3** (Scheme 1). Subsequent alcohol oxidation followed by reductive amination of the resulting aldehyde **4** with veliparib [6–7] provides iVeliparib-AP6. As a common practice for small-molecule library synthesis, the identity and the purity of the reaction products of this simple, 3-step process were analyzed by LC–MS.

**Figure 2:**
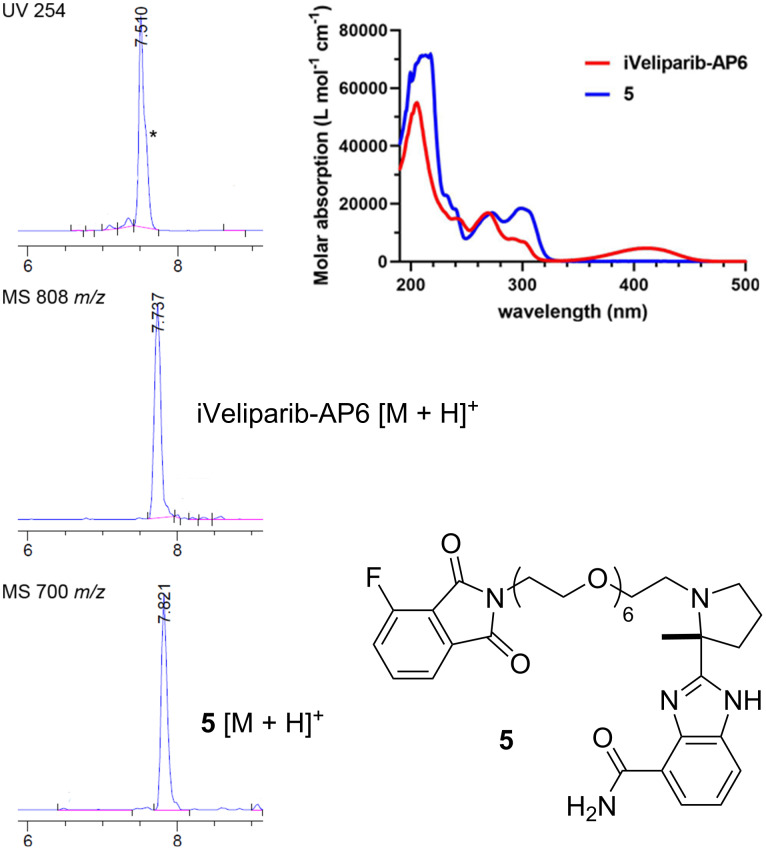
Identification of the cryptic impurity of iVeliparib. The UV trace of the initially purified iVeliparib-AP6 and the MS ion extraction traces of the desired product and the byproduct **5** on HPLC.

Whereas the HPLC traces of the reaction mixtures showed one major product peak, the final product carried a barely noticeable shoulder peak (Figure 2, marked with asterisk). A close examination of the UV profile indicated the presence of an impurity. Its removal requires repetitive prep-HPLC purifications that are inefficient and time-consuming. The isolated impurity lacks the characteristic fluorescent yellow color of pomalidomide or its close analogs, indicating that a side reaction unrelated to the S_N_Ar of the fluoride has occurred. ^1^H NMR and MS analyses suggested that phthalimide **5** was the byproduct formed through this series of transformations.

**Figure 3:**
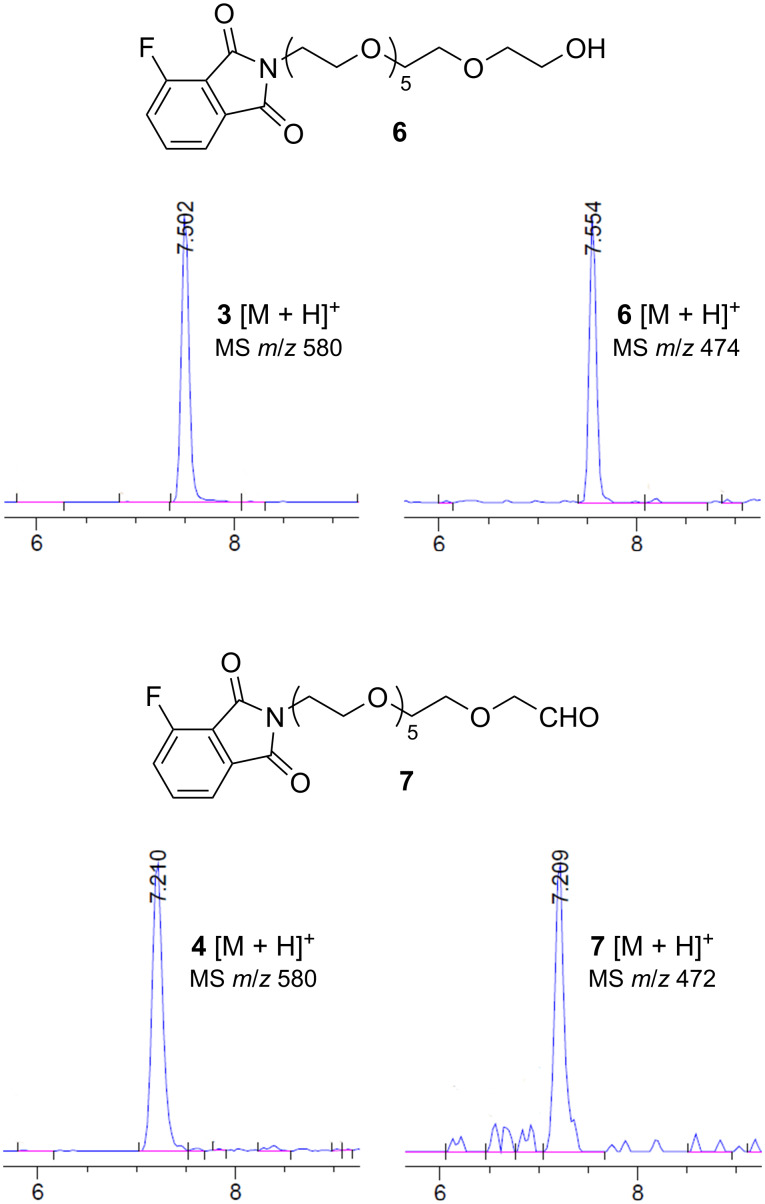
The structures of **6** and **7** and the MS ion extraction traces of **3**, **4**, **6**, and **7** from HPLC analysis of the reaction mixtures after purification by silica gel column chromatography.

Supporting this hypothesis, analysis of the reaction intermediates confirmed the presence of **6** that similarly co-eluted with **3** and **7** that completely overlapped with **4** on HPLC under our standard conditions (MeCN/0.1% TFA in water, 10%→60% 0→7 min, 60%→100% 7→10 min, 100% 10→15 min) (Figure 3). The identity of these impurities was further confirmed by independent synthesis of **5** using the same sequence of reactions starting from 3-fluorophthalic anhydride instead of **1**. Indeed, **5** is a white solid without UV absorption around 410 nm (Figure 2).

The formation of **6** originated from nucleophilic acyl substitution to displace the glutarimide in **1** by **2**. Interestingly, this side reaction was concentration dependent, giving various amounts of **6** under otherwise the same reaction conditions. For example, at 10–30 mM wherein most of the pilot studies were conducted, the ratio of **3** and **6** could be as low as ≈1:50 by ^1^H NMR and ≈1:10 by MS analysis. Under these conditions, the formation of **6** can evade detection if unaware of this issue. However, the amount of **6** can increase to as much as 20% when conducting the reaction at 0.1–0.3 M, the concentrations generally used for preparative work. Notably, because the S_N_Ar reaction of **1** proceeded slowly, 4-(dimethylamino)thalidomide also formed due to the decomposition of DMF. Whereas switching the solvent could prevent its formation, the accumulation of **7** remained an issue, giving no substantial improvement in the yield of **3** in, for example, NMP. In MeCN, glutarimide displacement became a major reaction. Because NMP was significantly more difficult to remove after the reaction, and 4-(dimethylamino)thalidomide could be separated from **3** easily, we opted to keep DMF as the solvent. We only detected by LC–MS a trace amount of the secondary byproduct derived from S_N_Ar of **6** or glutarimide displacement of **3** by **2**.

To understand the extent by which glutarimide displacement affects quality control analysis, we tested a series of amines and compared the retention times of **8** and **9** (Table 1). Rather surprisingly, the length of amino-PEG-OH had no effect on the relative retention time of **8** and **9**. The byproduct co-eluted with the desired product on HPLC even for the reaction of diethylene glycolamine and **1**. Capping the free hydroxy group with a methyl group improved the separation on HPLC marginally. However, incorporating a clickable propargyl group greatly benefited separation. Similar to the alcohol, amino-PEG5-acid also reacted with **1** to generate an inseparable byproduct. By contrast, the byproduct derived from Boc-protected amines has a significantly different retention time on HPLC and could be removed by regular silica gel chromatography.

**Table 1:** Effects of the length and functionalization of the PEG chain on the relative retention time of **8** and **9** on HPLC.

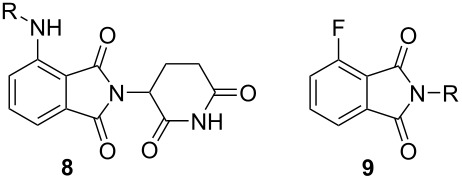		

Entry	R	Δ (rt **8**/**9**)^a^

1	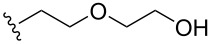	0.1 min
2	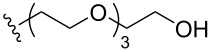	<0.1 min
3	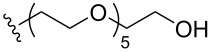	<0.1 min
4	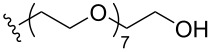	<0.1 min
5	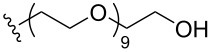	<0.1 min
6	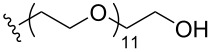	<0.1 min
7	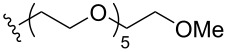	0.2 min
8	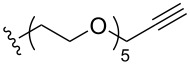	0.5 min
9	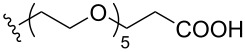	0.1 min
10	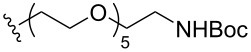	0.5 min
11	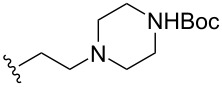	0.3 min

^a^Difference of the HPLC retention time for **8** and **9**.

**Scheme 2:**
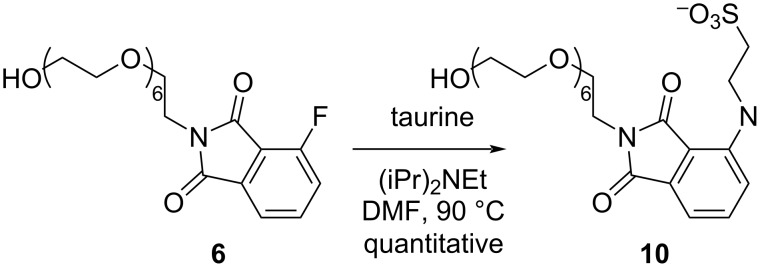
The conversion of byproduct **6** to **10** to facilitate its removal.

Because the formation of **5**‒**7** posts a significant purification challenge, we sought to develop a method to facilitate the elimination of this impurity. We first attempted scavenging **6** by solid-phase supported amines. Incubating a mixture of **3** and **6** with TentaGel S-NH_2_ in DMF led to a gradual decrease of **6** over four days. Whereas this method is applicable to removing **9** of different PEG-OH length (including **6** for *n* = 5), the decontamination process is slow and the costs are high. Additionally, it could not remove the trace amount of the residual impurity, potentially due to inefficient diffusion of the substrate into the resin at low concentrations. We next sought to use soluble amines to enable an easy separation of the byproduct. Pleasingly, reacting the mixture of **3** and **6** with taurine converted **6** to sulfonate **10** (Scheme 2) that can be removed by a simple aqueous wash with sodium bicarbonate. Whereas taurine may also react with **3**, the overall yield of **3** was not affected significantly. In a typical run, we isolated a 3:1 mixture of **3** and **6** in 54% combined yield and obtained pure **3** in 41% yield after taurine treatment. We did not observe any byproduct corresponding to glutarimide displacement of **3** by taurine. Using this method, we could easily prepare 100 mg of iVeliparib-AP6 with <1% impurity based on ^1^H NMR and MS analysis.

**Figure 4:**
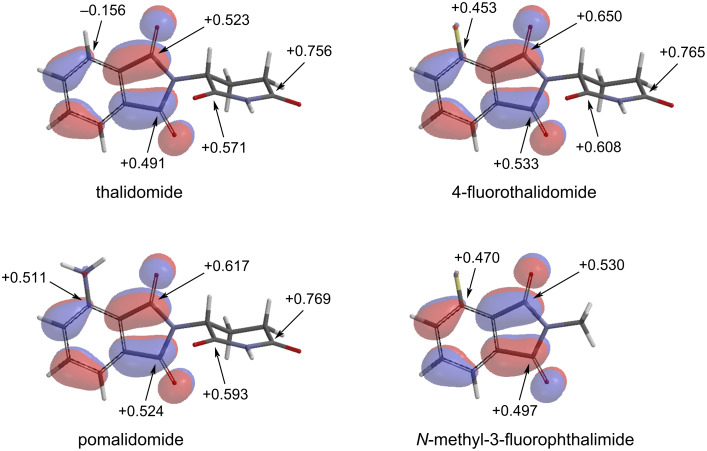
The LUMO and electrostatic charges of thalidomide and its derivatives by DFT calculation at the ωB97X-D/6-311+G** level.

The mechanism by which glutarimide displacement competes with S_N_Ar merits some discussion. Thalidomide is well known for its configurational instability and racemizes rapidly [8]. However, equally important but much less appreciated is its hydrolytic instability, where the cleavage of the phthalimide and glutarimide rings comprises the major metabolic pathways [9–10]. Computational analysis confirms that C1, C3, C2’, and C4’ are the most electrophilic sites of thalidomide (Figure 4). As expected, introducing a fluorine atom to C4 activates it toward S_N_Ar. Although C2’ and C4’ remain the most electropositive sites, we could not isolate any byproducts corresponding to reactions with glutarimide. The preferred nucleophilic reaction at C1, C3, and C4 can be explained by the fact that the LUMO of **1** resides entirely on phthalimide. The low S_N_Ar reaction rate may also be explained by C4 being located near the node of the LUMO. However, S_N_Ar is still favored over glutarimide displacement potentially because the negative charge in the corresponding intermediate is stabilized by an extended conjugation system. In contrast, the negative charge in the carbonyl addition intermediate is stabilized by an oxygen atom only. As such, the erosion of **3** by taurine was minimal.

## Conclusion

Nucleophilic aromatic substitution of 4-fluorothalidomide (**1**) has provided a convenient entry to the IMiD class of PROTAC molecules. Although the yield of the desired product is generally modest, it remains highly popular because of the convenience and the modularity of this method. When reacting **1** with an amino-PEG-OH, the purification of the reaction mixture is particularly difficult as a major byproduct co-elutes with the desired product even on HPLC. Importantly, the impurity issue can persist throughout the remainder of the synthesis if not addressed directly. To our knowledge, there has been no discussion regarding the identity of this impurity. We show herein that nucleophilic acyl substitution competes with aromatic substitution to displace the glutarimide. Introducing a sulfonate to the byproduct by reacting it with taurine allows for easy decontamination. This method may facilitate the purification of other similar reactions and improve the quality of related pomalidomide derivatives.

## Supporting Information

File 1 Computational details, general experimental information, synthetic procedures, compound characterization data, and copies of NMR spectra.

## Data Availability

All data that supports the findings of this study is available in the published article and/or the supporting information of this article.
