# Supplementary material for: Identification and removal of a cryptic impurity in pomalidomide-PEG based PROTAC
Source: Beilstein J Org Chem. 2025 Feb 18;21:407–11. doi: 10.3762/bjoc.21.28 (PMC11849548; doi:10.3762/bjoc.21.28)
Supplement: File 1 — Computational details, general experimental information, synthetic procedures, compound characterization data, and copies of NMR spectra. [file Beilstein_J_Org_Chem-21-407-s001.pdf]

BEILSTEIN JOURNAL OF ORGANIC CHEMISTRY

Supporting Information

for

Identification and removal of a cryptic impurity in pomalidomide-PEG based PROTAC

Bingnan Wang, Yong Lu and Chuo Chen

Beilstein J. Org. Chem. **2025,** *21,* 407–411. doi:10.3762/bjoc.21.28

Computational details, general experimental information, synthetic procedures, compound characterization data, and copies of NMR spectra

License and Terms: This is a supporting information file under the terms of the Creative Commons Attribution License (https://creativecommons.org/licenses/by/4.0). Please note that the reuse, redistribution and reproduction in particular requires that the author(s) and source are credited and that individual graphics may be subject to special legal provisions.
The license is subject to the *Beilstein Journal of Organic Chemistry* terms and conditions: (https://www.beilstein-journals.org/bjoc/terms)

Table of contents

Computational details S1

General information S6

Synthetic procedures S7

NMR spectra S11

Computational details

The computations were performed on Spartan '18 (Wavefunction, Inc. Irvine, CA) suite of programs using DFT at the ωB97X-D/6-311+G** level wherein the natural charges were obtained from a natural population analysis and the electrostatic charges calculated based on the CHELP algorithm with a standard shell thickness of 5.5 au.

Thalidomide

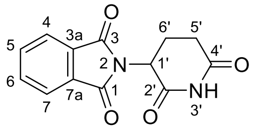

Atom label	Natural charge	Electrostatic charge
C1	0.727	0.491
C3	0.728	0.523
C3a	-0.116	-0.020
C4	-0.162	-0.156
C5	-0.184	-0.106
C6	-0.182	-0.096
C7	-0.162	-0.168
C7a	-0.115	0.014
C1'	-0.111	0.271
C2'	0.718	0.571
C4'	0.718	0.756
C5'	-0.485	-0.539
C6'	-0.404	-0.162
H4	0.234	0.160
H5	0.217	0.137
H6	0.217	0.136
H7	0.234	0.160
H1'	0.262	0.078
H5'a	0.233	0.172
H5'e	0.246	0.200
H6'a	0.234	0.133
H6'e	0.222	0.108
HN3'	0.426	0.390
N2	-0.538	-0.448
N3'	-0.672	-0.665
O1	-0.565	-0.453
O3	-0.564	-0.471
O2'	-0.579	-0.478
O4'	-0.581	-0.541

4-Fluorothalidomide

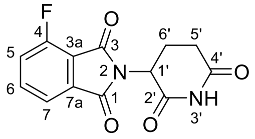

Atom label	Natural charge	Electrostatic charge
C1	0.724	0.533
C3	0.731	0.650
C3a	-0.188	-0.271
C4	0.465	0.453
C5	-0.253	-0.348
C6	-0.163	-0.003
C7	-0.181	-0.248
C7a	-0.090	0.066
C1'	-0.111	0.183
C2'	0.718	0.608
C4'	0.719	0.765
C5'	-0.485	-0.559
C6'	-0.403	-0.137
F4	-0.323	-0.213
H5	0.235	0.205
H6	0.221	0.138
H7	0.238	0.175
H1'	0.263	0.095
H5'a	0.234	0.182
H5'e	0.247	0.202
H6'a	0.233	0.135
H6'e	0.223	0.112
HN3'	0.426	0.389
N2	-0.537	-0.490
N3'	-0.672	-0.670
O1	-0.562	-0.458
O3	-0.547	-0.471
O2'	-0.579	-0.484
O4'	-0.580	-0.540

Pomalidomide

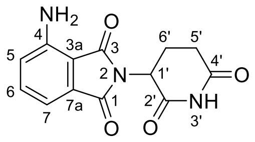

Atom label	Natural charge	Electrostatic charge
C1	0.729	0.524
C3	0.731	0.617
C3a	-0.195	-0.298
C4	0.230	0.511
C5	-0.242	-0.375
C6	-0.156	-0.008
C7	-0.211	-0.272
C7a	-0.083	0.067
C1'	-0.111	0.192
C2'	0.718	0.593
C4'	0.719	0.769
C5'	-0.484	-0.570
C6'	-0.403	-0.106
H5	0.212	0.186
H6	0.215	0.133
H7	0.233	0.171
H1'	0.261	0.090
H5'a	0.232	0.179
H5'e	0.246	0.201
H6'a	0.234	0.125
H6'e	0.222	0.102
HN3'	0.425	0.389
HN4a	0.389	0.395
HN4b	0.418	0.398
N2	-0.538	-0.459
N3'	-0.672	-0.666
O1	-0.566	-0.460
O3	-0.605	-0.510
O2'	-0.579	-0.489
O4'	-0.583	-0.545
N4	-0.787	-0.884

N-Methyl-3-fluorophthalimide

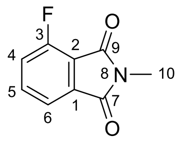

Atom label	Natural charge	Electrostatic charge
C1	-0.092	-0.034
C2	-0.189	-0.266
C3	0.460	0.470
C4	-0.255	-0.347
C5	-0.166	-0.034
C6	-0.186	-0.184
C7	0.711	0.497
C9	0.715	0.530
C10	-0.373	-0.652
F3	-0.325	-0.219
H4	0.234	0.204
H5	0.220	0.141
H6	0.237	0.164
H10a	0.212	0.223
H10b	0.209	0.223
H10c	0.228	0.237
N8	-0.529	-0.040
O7	-0.565	-0.468
O9	-0.547	-0.445

General information

All solvents for the synthesis were purified by passing commercially available pre-dried, oxygen-free formulations through activated alumina columns. Reactions were monitored by TLC or LC–MS and the products were purified by flash column chromatography unless otherwise mentioned. NMR spectra were recorded on a Bruker AN400 or AN600 instrument. The chemical shifts of the ^{1}H NMR spectra are reported in ppm (δ) relative to the ^{1}H signals in the solvent ($CDCl_3$: δ 7.26 ppm; MeOD: δ 3.31 ppm) and the multiplicities are presented as follows: s = singlet, d = doublet, t = triplet, q = quartet, m = multiplet. LC-MS was performed on an Agilent 1260 HPLC machine coupled to a 6120 single quadrupole MS detector using an Agilent Eclipse XDB-C18 5 µm 4.6 × 150 mm column. HRMS was performed on a Sciex TripleTOF 6600 mass spectrometer coupled to Shimadzu HPLC (Nexera X2 LC-30AD).

Synthetic procedures

Synthesis of iVeliparib-AP6

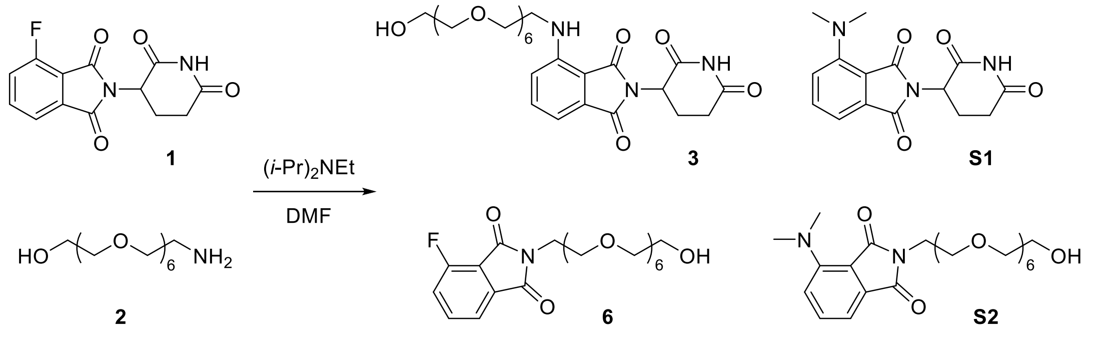

To a 4 mL clear glass vial charged with **1** (85 mg, 0.308 mmol, 1.0 equiv) in *N*,*N*-dimethylformamide (1 mL) was added **2** (71 mg, 0.308 mmol, 1.0 equiv) and *N*,*N*-diisopropylethylamine (40 mg, 0.308 mmol, 1.0 equiv, 54 μL) at 23 °C. After stirring at 90 °C overnight, the solution was concentrated. The residue was diluted with methylene chloride (10 mL) and washed with saturated aqueous sodium bicarbonate solution (10 mL). The layers were separated, and the aqueous layer was extracted with methylene chloride (3 × 10 mL). The combined organic layers were dried with anhydrous sodium sulfate, concentrated, and purified by flash column chromatography on silica gel (methanol/methylene chloride 1:10) to afford a mixture of **3** and **6** (92 mg, 54%, 3:1 by ^{1}H NMR) as a yellow oil, **1** (14 mg, 16%) as a white solid, **S1** (18 mg, 19%) as a yellow oil, and **S2** (13 mg, 8%) as a yellow oil.

To a 4 mL clear glass vial charged with the resulting mixture of **3** and **6** in *N*,*N*-dimethylformamide (1 mL) was added taurine (20 mg, 0.158 mmol, 1.0 equiv) and *N*,*N*-diisopropylethylamine (41 mg, 0.316 mmol, 2.0 equiv, 30 μL) at 23 °C. After stirring at 90 °C for 30 h, the solution was concentrated. The residue was diluted with methylene chloride (5 mL) and washed with saturated aqueous sodium bicarbonate (5 mL). The layers were separated, and the aqueous layer was extracted with methylene chloride (3 × 5 mL). The combined organic layers were dried with anhydrous sodium sulfate and concentrated to afford **3** (73 mg, 41%) as a yellow oil [*Nat. Chem. Biol.* **2019**, *15*, 1223].

S1: R_f = 0.35 (silica gel, methanol/methylene chloride 1:10); ^{1}H NMR (400 MHz, $CDCl_3$) δ 8.56 (s, 1H), 7.70 (t, *J* = 7.7 Hz, 1H), 7.59 (d, *J* = 7.1 Hz, 1H), 7.52 (d, *J* = 8.2 Hz, 1H), 5.01 (dd, *J* = 11.9, 5.2 Hz, 1H), 3.25 (s, 6H), 2.99 – 2.69 (m, 3H), 2.22 – 2.09 (m, 1H); ^{13}C NMR (150 MHz, $CDCl_3$) δ 171.8, 168.5, 166.7, 166.2, 146.2, 136.3, 134.5, 125.2, 119.1, 118.1, 49.5, 44.4 (2C), 31.4, 22.6; MS (ESI): calculated for $C_{15}H_{16}N_3O_4$ $(M+H)^+$ 302.1, found 302.1.

S2: R_f = 0.25 (silica gel, methanol/methylene chloride 1:10); ^{1}H NMR (400 MHz, $CDCl_3$) δ 7.57 (t, *J* = 7.7 Hz, 1H), 7.51 – 7.35 (m, 2H), 3.87 (t, *J* = 5.9 Hz, 2H), 3.75 – 3.57 (m, 28H), 3.16 (s, 6H); ^{13}C NMR (150 MHz, $CDCl_3$) δ 167.3, 161.9, 136.2, 135.4, 135.1, 124.4, 117.7, 115.9, 72.6, 70.71, 70.67, 70.65 (3C), 70.62 (2C), 70.60, 70.4, 70.1, 68.0, 61.8, 44.1 (2C), 37.4; MS (ESI): calculated for $C_{24}H_{39}N_2O_9$ $(M+H)^+$ 499.3, found 499.3.

3: R_f = 0.20 (silica gel, methanol/methylene chloride 1:10); ^{1}H NMR (600 MHz, $CDCl_3$) δ 8.90 (s, 1H), 7.46 (dd, *J* = 8.5, 7.2 Hz, 1H), 7.06 (d, *J* = 7.2 Hz, 1H), 6.89 (d, *J* = 8.5 Hz, 1H), 4.89 (dd, *J* = 12.3, 5.4 Hz, 1H), 3.69 (dd, *J* = 7.1, 4.1 Hz, 4H), 3.63 (dd, *J* = 10.4, 4.6 Hz, 20H), 3.59 – 3.56 (m, 2H), 3.44 (q, *J* = 5.5 Hz, 2H), 2.87 – 2.80

(m, 1H), 2.82 – 2.66 (m, 2H), 2.12 – 2.05 (m, 1H); ^{13}C NMR (150 MHz, $CDCl_3$) δ 171.5, 169.3, 168.7, 167.8, 146.9, 136.1, 132.6, 116.9, 111.8, 110.4, 72.7, 70.8, 70.70, 70.65, 70.64, 70.61 (2C), 70.57, 70.56, 70.51, 70.3, 69.5, 61.7, 49.0, 42.5, 31.6, 22.9; HRMS (ESI): calculated for $C_{27}H_{40}N_3O_{11}$ $(M+H)^+$ 582.2657, found 582.2677.

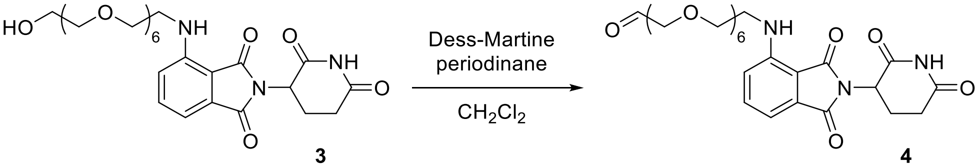

To a 12 mL clear glass vial charged with **3** (525 mg, 0.90 mmol, 1.0 equiv) in methylene chloride (10 mL) was added Dess–Martin periodinane (594 mg, 1.4 mmol, 1.5 equiv) at 0 °C. After stirring at 23 °C for 2 h, the reaction was quenched with saturated aqueous sodium bicarbonate (5 mL) and saturated aqueous sodium thiosulfate (5 mL). The layers were separated, and the aqueous layer was extracted with methylene chloride (3 × 10 mL). The combined organic layers were dried with anhydrous sodium sulfate, concentrated, and purified by flash column chromatography on silica gel (methanol/methylene chloride 1:10) to afford **4** (290 mg, 55%) as a yellow oil [*Nat. Chem. Biol.* **2019**, *15*, 1223]: R_f = 0.25 (silica gel, methanol/methylene chloride 1:10); ^{1}H NMR (400 MHz, $CDCl_3$) δ 9.71 (s, 1H), 8.50 (s, 1H), 7.48 (dd, *J* = 8.5, 7.1 Hz, 1H), 7.09 (d, *J* = 7.1 Hz, 1H), 6.91 (d, *J* = 8.5 Hz, 1H), 6.48 (brs, 1H), 4.90 (dd, *J* = 12.0, 5.5 Hz, 1H), 4.15 (s, 2H), 3.78 – 3.59 (m, 22H), 3.46 (t, *J* = 5.3 Hz, 2H), 2.92 – 2.66 (m, 3H), 2.16 – 2.06 (m, 1H); ^{13}C NMR (150 MHz, $CDCl_3$) δ 201.1, 171.5, 169.4, 168.6, 167.8, 146.9, 136.1, 132.6, 116.9, 111.7, 110.4, 72.7, 71.3, 70.82, 70.77, 70.72, 70.68, 70.64 (2C), 70.55, 70.3, 69.5, 61.7, 49.0, 42.5, 31.5, 22.9; HRMS (ESI): calculated for $C_{27}H_{38}N_3O_{11}$ $(M+H)^+$ 580.2501, found 580.2518.

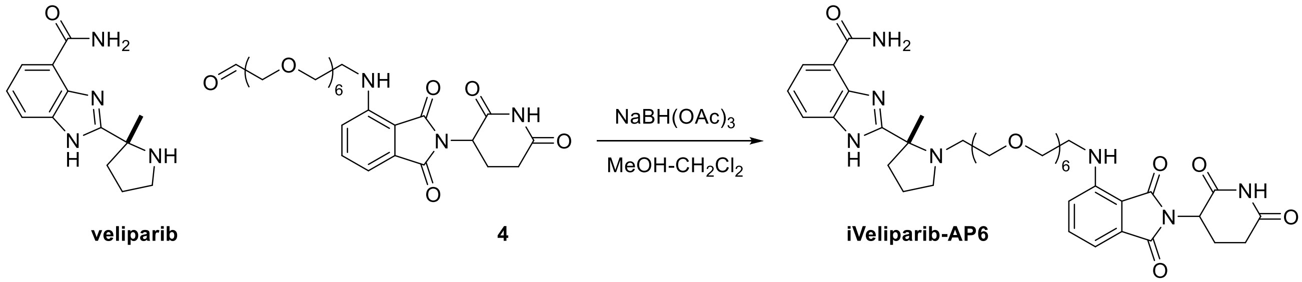

To a 4 mL clear glass vial charged with **4** (58 mg, 0.10 mmol, 1.0 equiv) in a mixture of methylene chloride (1 mL) and methanol (1 mL) was added veliparib (24 mg, 0.10 mmol, 1.0 equiv) followed by sodium triacetoxyborohydride (42 mg, 0.20 mmol, 2.0 equiv) at 0 °C. After stirring at 23 °C overnight, the solution was concentrated and purified by flash column chromatography on silica gel (methanol/methylene chloride 1:10) to afford iVeliparib-AP6 (44 mg, 54%) as a yellow oil [*Nat. Chem. Biol.* **2019**, *15*, 1223]: R_f = 0.20 (silica gel, methanol/methylene chloride 1:10); ^{1}H NMR (400 MHz, MeOD) δ 8.01 (d, *J* = 7.6 Hz, 1H), 7.77 (d, *J* = 8.1 Hz, 1H), 7.54 – 7.48 (m, 1H), 7.43 (t, *J* = 7.8 Hz, 1H), 7.07 – 6.98 (m, 2H), 5.03 (dd, *J* = 12.4, 5.4 Hz, 1H), 4.08 (brs, 1H), 3.85 (s, 2H), 3.73 – 3.33 (m, 28H), 2.92 – 2.78 (m, 1H), 2.79 – 2.63 (m, 2H), 2.59 – 2.22 (m, 4H), 2.10 (tdd, *J* = 8.3, 5.6, 2.8 Hz, 1H), 1.90 (s, 3H); ^{13}C NMR (150 MHz, MeOD) δ 174.7, 171.6, 170.6, 169.5, 169.2, 148.1, 140.8, 137.3, 136.9, 133.7, 125.2, 124.6, 123.2, 118.3, 117.5, 114.5, 112.0, 111.1, 71.8, 71.53, 71.49, 71.47, 71.44,

71.42, 71.40, 71.3, 71.2, 70.5, 66.6, 53.2, 52.6, 50.2, 43.2, 38.9, 32.2, 23.8, 21.7, 18.3; HRMS (ESI): calculated for $C_{40}H_{54}N_7O_{11}$ $(M+H)^+$ 808.3876, found 808.3911.

*Synthesis of impurity **5***

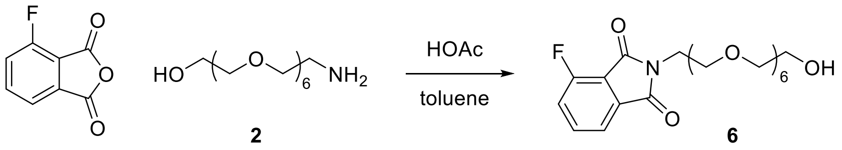

To a 4 mL clear glass vial charged with 3-fluorophthalic anhydride (17 mg, 0.10 mmol, 1.0 equiv) in a mixture of acetic acid (0.2 mL) and toluene (0.4 mL) was added **2** (33 mg, 0.10 mmol, 1.0 equiv) at 23 °C. After stirring at 80 °C overnight, the solution was concentrated. The residue was diluted with methylene chloride (5 mL) and washed with saturated aqueous sodium bicarbonate (5 mL). The layers were separated, and the aqueous layer was extracted with methylene chloride (3 × 5 mL). The combined organic layers were dried with anhydrous sodium sulfate, concentrated, and purified with flash column chromatography on silica gel (methanol/methylene chloride 1:10) to afford **6** (18 mg, 38%) as a colorless oil: R_f = 0.20 (silica gel, methanol/methylene chloride 1:10); ^{1}H NMR (400 MHz, $CDCl_3$) δ 7.68 (ddd, *J* = 8.3, 7.3, 4.3 Hz, 1H), 7.61 (d, *J* = 7.2 Hz, 1H), 7.33 (td, *J* = 8.5, 0.9 Hz, 1H), 3.83 (t, *J* = 5.8 Hz, 2H), 3.74 – 3.49 (m, 27H), 3.04 (s, 1H); ^{13}C NMR (150 MHz, $CDCl_3$) δ 167.1 (d, *J* = 2.8 Hz), 164.9, 157.5 (d, *J* = 265.6 Hz), 136.6 (d, *J* = 7.6 Hz), 134.3, 122.4 (d, *J* = 19.7 Hz), 119.5 (d, *J* = 3.5 Hz), 117.8 (d, *J* = 12.5 Hz), 72.6, 70.6, 70.52, 70.49 (3C), 70.47 (2C), 70.45, 70.2, 70.0, 67.7, 61.6, 37.4; ^{19}F NMR (565 MHz, $CDCl_3$) δ -113.1; HRMS (ESI): calculated for $C_{22}H_{33}FNO_9$ $(M+H)^+$ 474.2134, found 474.2147.

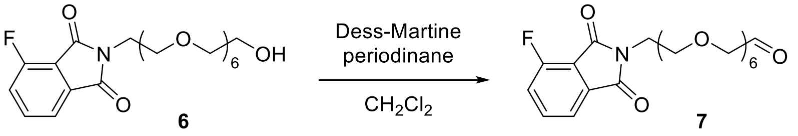

To a 4 mL clear glass vial charged with **6** (18 mg, 0.038 mmol, 1.0 equiv) in methylene chloride (1 mL) was added Dess–Martin periodinane (48 mg, 0.11 mmol, 3.0 equiv) at 0 °C. After stirring at 23 °C for 2 h, the solution was quenched with saturated aqueous sodium bicarbonate (1 mL) and saturated aqueous sodium thiosulfate (1 mL). The layers were separated, and the aqueous layer was extracted with methylene chloride (3 × 5 mL). The combined organic layers were dried with anhydrous sodium sulfate, concentrated, and purified by flash column chromatography on silica gel (methanol/methylene chloride 1:10) to afford **7** (9.0 mg, 50%) as a colorless oil: R_f = 0.25 (silica gel, methanol/methylene chloride 1:10); ^{1}H NMR (600 MHz, $CDCl_3$) δ 9.72 (t, *J* = 0.9 Hz, 1H), 7.71 (ddd, *J* = 8.2, 7.3, 4.3 Hz, 1H), 7.66 (dd, *J* = 7.3, 0.8 Hz, 1H), 7.37 (td, *J* = 8.5, 0.9 Hz, 1H), 4.16 (d, *J* = 0.8 Hz, 1H), 3.88 (t, *J* = 5.8 Hz, 2H), 3.75 – 3.68 (m, 5H), 3.66 – 3.57 (m, 19H); ^{13}C NMR (150 MHz, $CDCl_3$) δ 201.1, 167.1 (d, *J* = 2.7 Hz), 165.0, 157.5 (d, *J* = 265.6 Hz), 136.6 (d, *J* = 7.4 Hz), 134.4, 122.4 (d, *J* = 19.9 Hz), 119.5 (d, *J* = 3.7 Hz), 117.9 (d, *J* = 12.4 Hz), 71.2, 70.8, 70.65, 70.61, 70.57, 70.55 (3C), 70.53 (2C), 70.0, 67.8, 37.4; ^{19}F NMR (376 MHz, $CDCl_3$) δ -113.0; HRMS (ESI): calculated for $C_{22}H_{31}FNO_9$ $(M+H)^+$ 472.1977, found 472.1973.

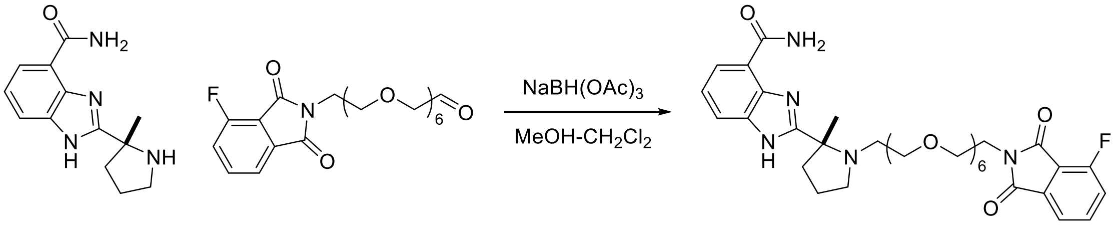

veliparib **7** **5**

To a 4 mL clear glass vial charged with **7** (9.0 mg, 0.019 mmol, 1.0 equiv) in a mixture of methanol (0.5 mL) and methylene chloride (0.5 mL) was added veliparib (4.6 mg, 0.019 mmol, 1.0 equiv) followed by sodium triacetoxyborohydride (8.1 mg, 0.038 mmol, 2.0 equiv) at 0 °C. After stirring at 23 °C overnight, the solution was concentrated and purified by preparative TLC on silica gel (methanol/methylene chloride 1:10) to afford **5** (8.2 mg, 62%) as a colorless oil: R_f = 0.20 (silica gel, methanol/methylene chloride 1:10); ^{1}H NMR (400 MHz, MeOD) δ 7.86 (d, *J* = 7.6 Hz, 1H), 7.79 (ddd, *J* = 8.4, 7.3, 4.4 Hz, 1H), 7.71 (dd, *J* = 8.0, 1.1 Hz, 1H), 7.65 (d, *J* = 7.3 Hz, 1H), 7.47 (t, *J* = 8.8 Hz, 1H), 7.28 (t, *J* = 7.8 Hz, 1H), 3.82 (t, *J* = 5.7 Hz, 2H), 3.73 – 3.40 (m, 29H), 2.90 – 2.77 (m, 2H), 2.69 (dt, *J* = 13.3, 4.0 Hz, 1H), 2.27 – 2.14 (m, 1H), 2.12 – 1.99 (m, 3H), 1.59 (s, 3H); ^{13}C NMR (150 MHz, MeOD) δ 170.5, 168.5 (d, *J* = 2.8 Hz), 166.3, 158.8 (d, *J* = 263.4 Hz), 138.2 (d, *J* = 7.7 Hz), 135.7, 123.7, 123.4 (d, *J* = 20.1 Hz), 122.9, 120.5 (d, *J* = 3.4 Hz), 118.9 (d, *J* = 12.6 Hz), 71.53, 71.49, 71.47 (2C), 71.44 (2C), 71.42, 71.40, 71.3, 71.2, 70.5, 68.7, 65.8, 52.3, 50.9, 42.0, 38.5, 23.1, 18.4; ^{19}F NMR (565 MHz, MeOD) δ −116.1; HRMS (ESI): calculated for $C_{35}H_{47}FN_5O_9$ $(M+H)^+$ 700.3352, found 700.3351.

General procedure for comparing the retention times of the nucleophilic aromatic substitution reaction products

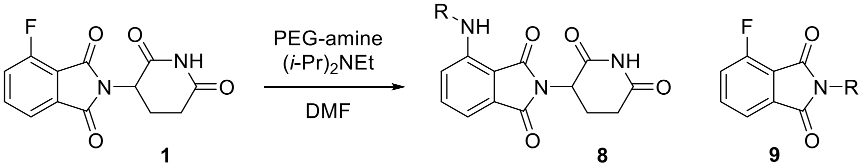

1 **8** **9**

To a 4 mL clear glass vial charged with **1** (28 mg, 0.10 mmol, 1.0 equiv) in *N*,*N*-dimethylformamide (1 mL) was added PEG-amine (0.10 mmol, 1.0 equiv) and *N*,*N*-diisopropylethylamine (26 mg, 0.20 mmol, 1.0 equiv, 35 μL) at 23 °C. After stirring at 90 °C overnight, the reaction mixture was anlalyzed by LC–MS using the ion extraction method to compare the retention times of **8** and **9**.

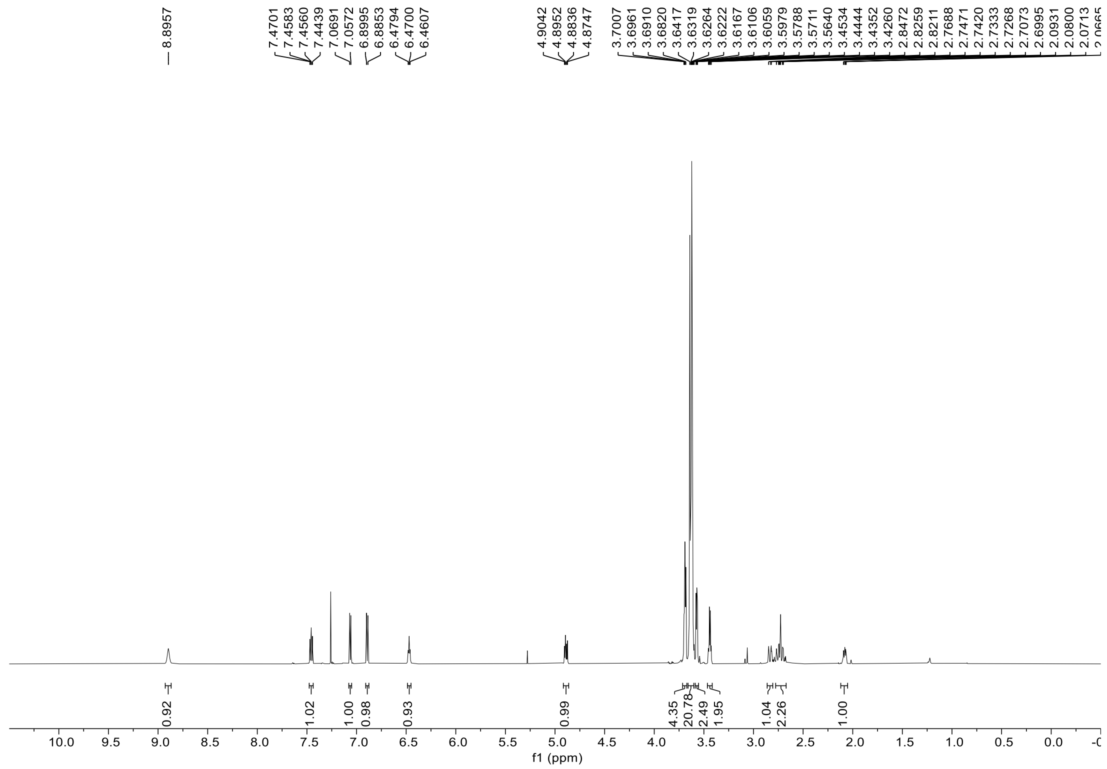

^{1}H NMR spectrum of **3** (600 MHz, $CDCl_3$)

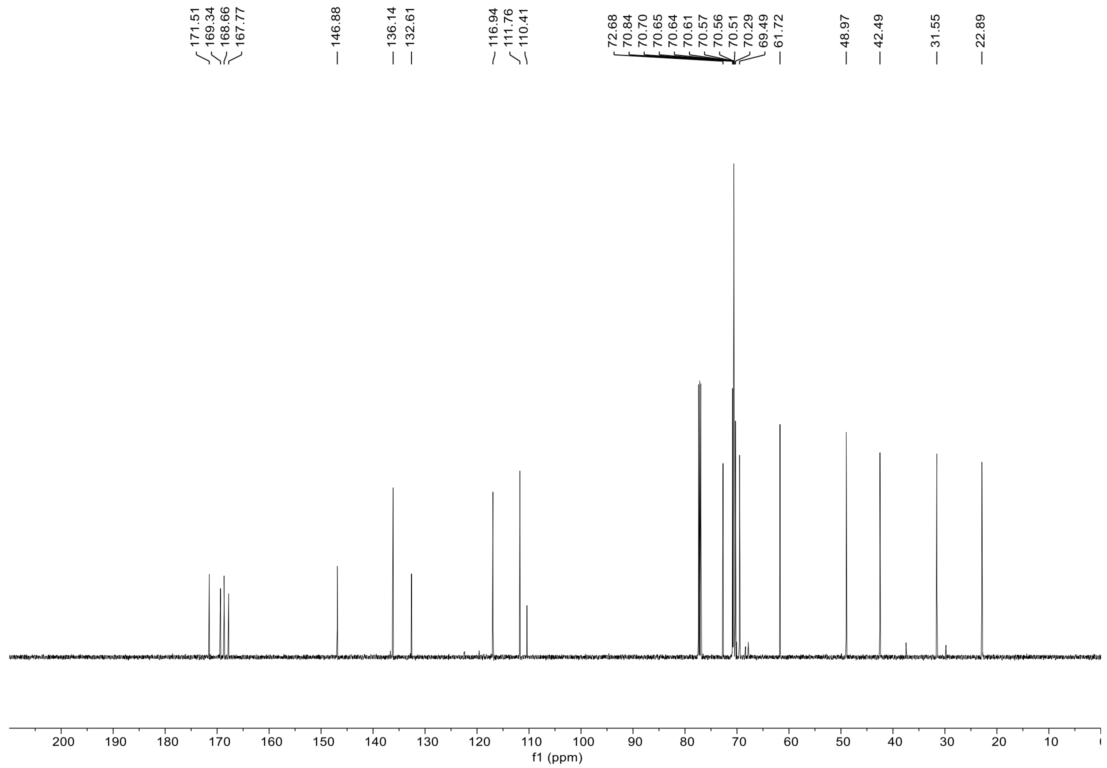

^{13}C NMR spectrum of **3** (150 MHz, $CDCl_3$)

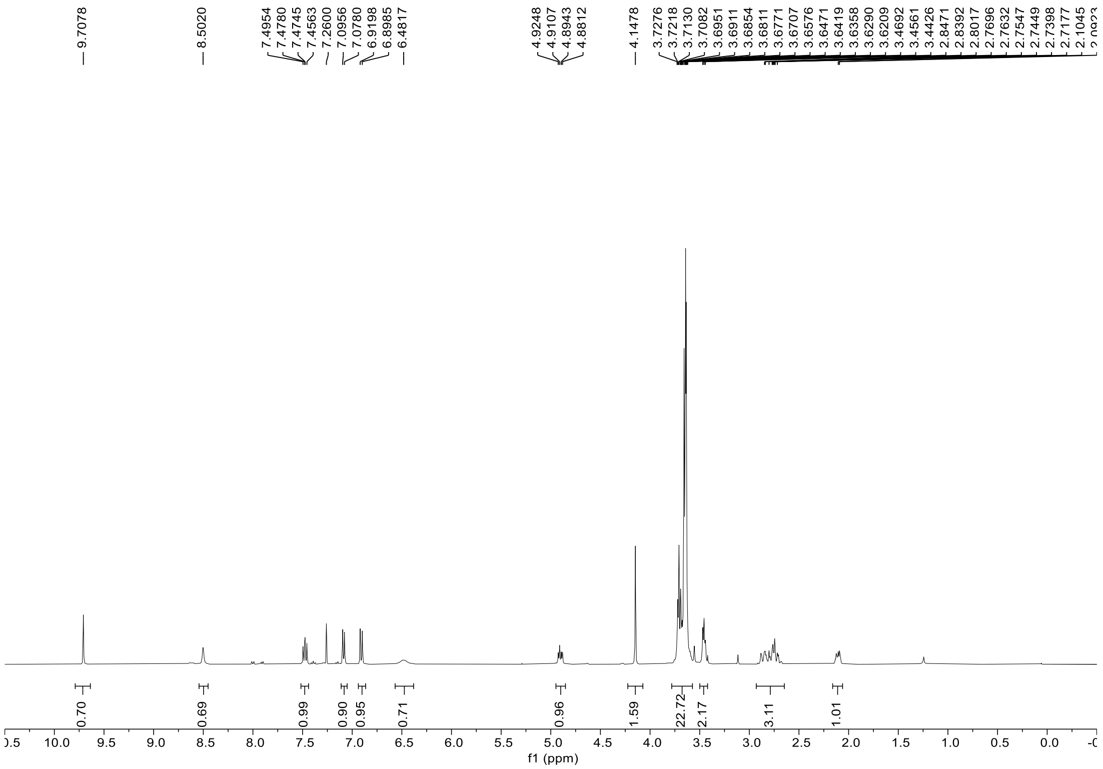

^{1}H NMR spectrum of **4** (400 MHz, $CDCl_3$)

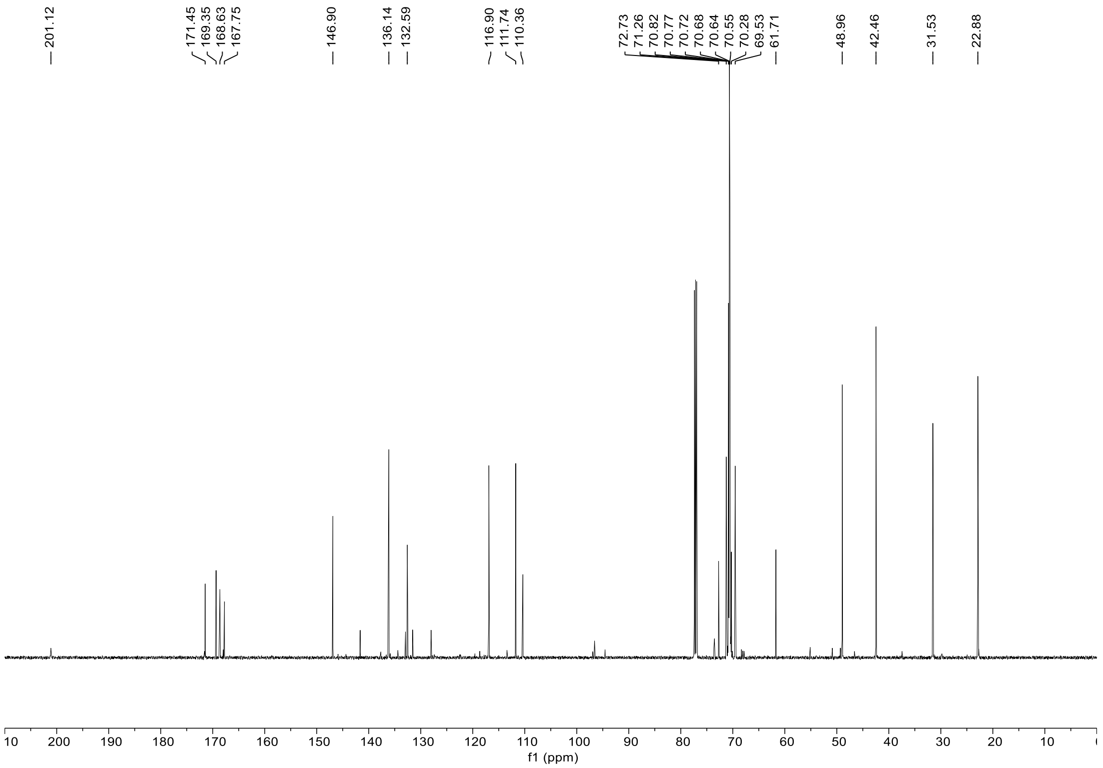

^{13}C NMR spectrum of **4** (150 MHz, $CDCl_3$)

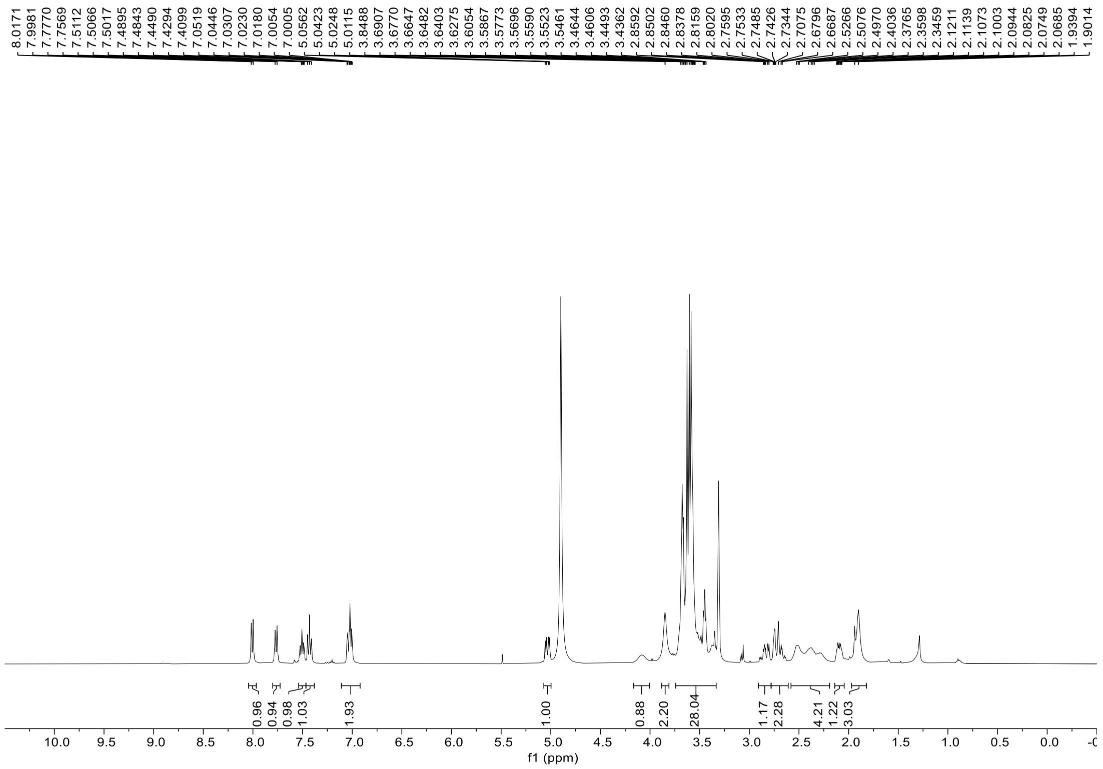

^{1}H NMR spectrum of iVeliparib-AP6 (400 MHz, CD_3OD)

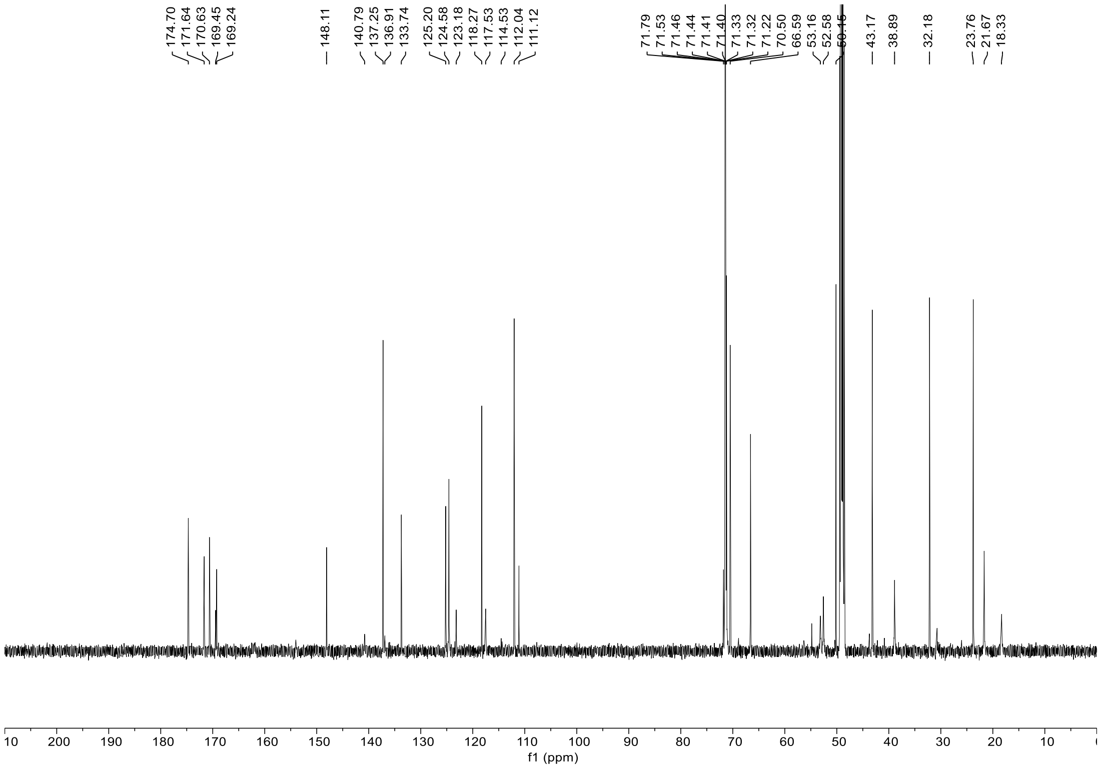

^{13}C NMR spectrum of iVeliparib-AP6 (150 MHz, CD_3OD)

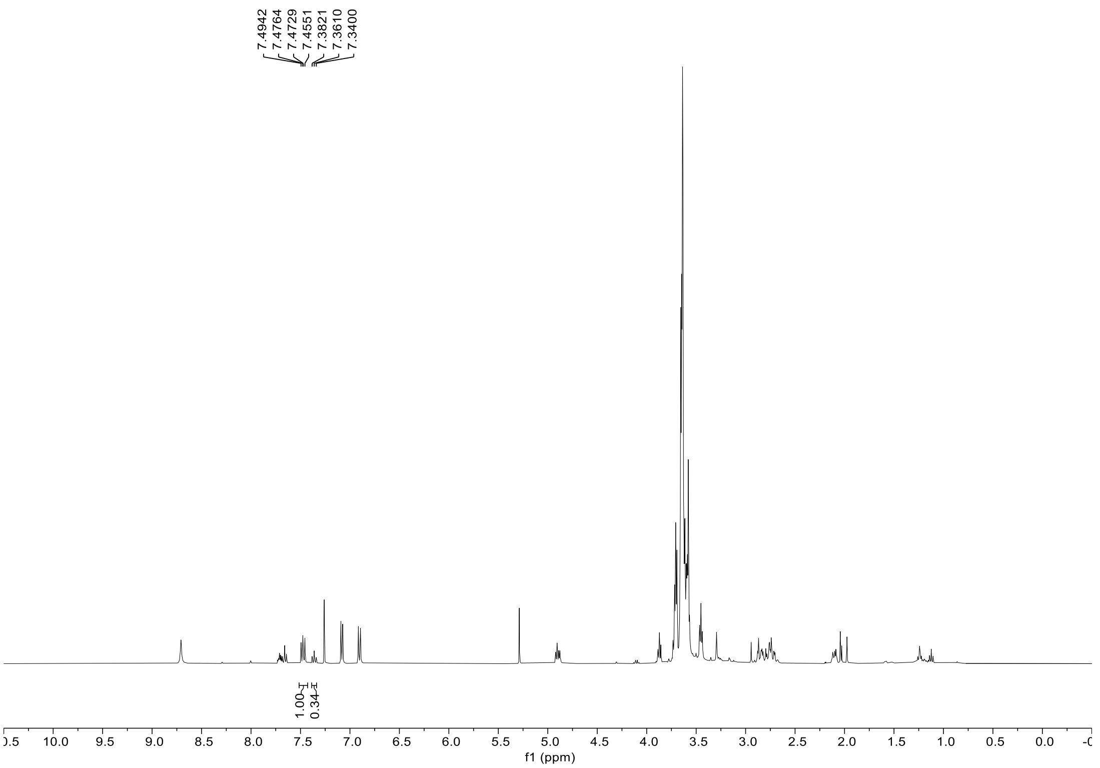

^{1}H NMR spectrum of the mixture of **3** and **6** (400 MHz, $CDCl_3$)

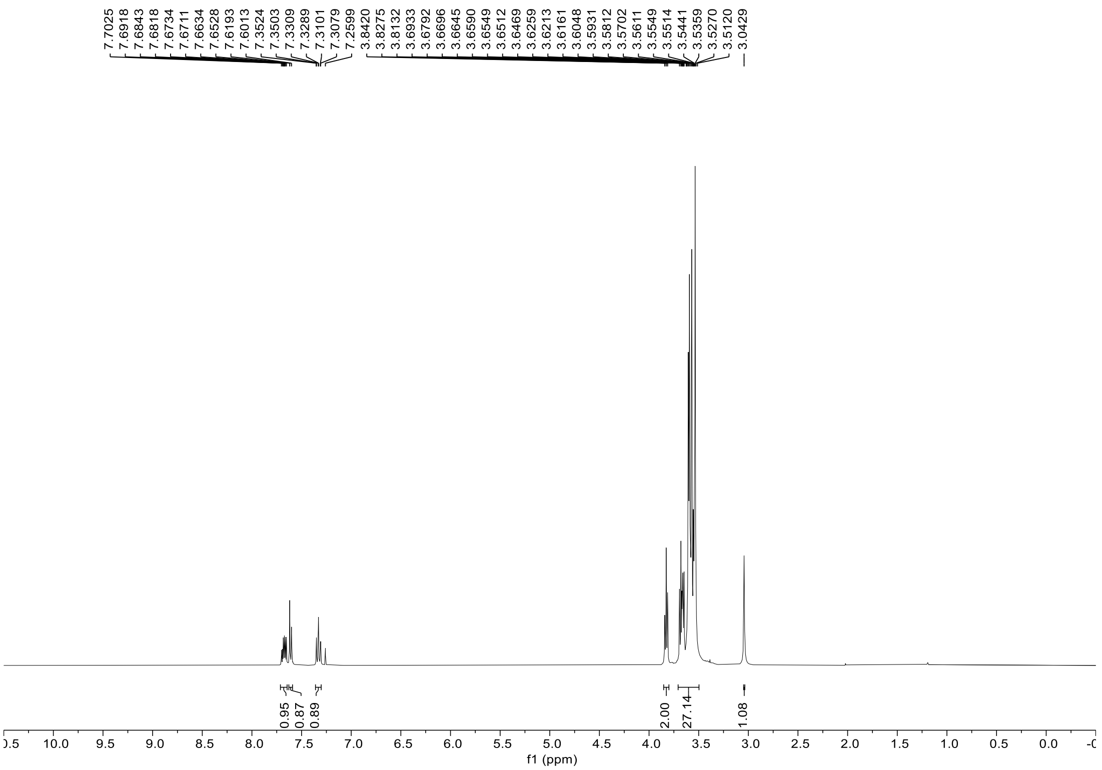

^{1}H NMR spectrum of **6** (600 MHz, $CDCl_3$)

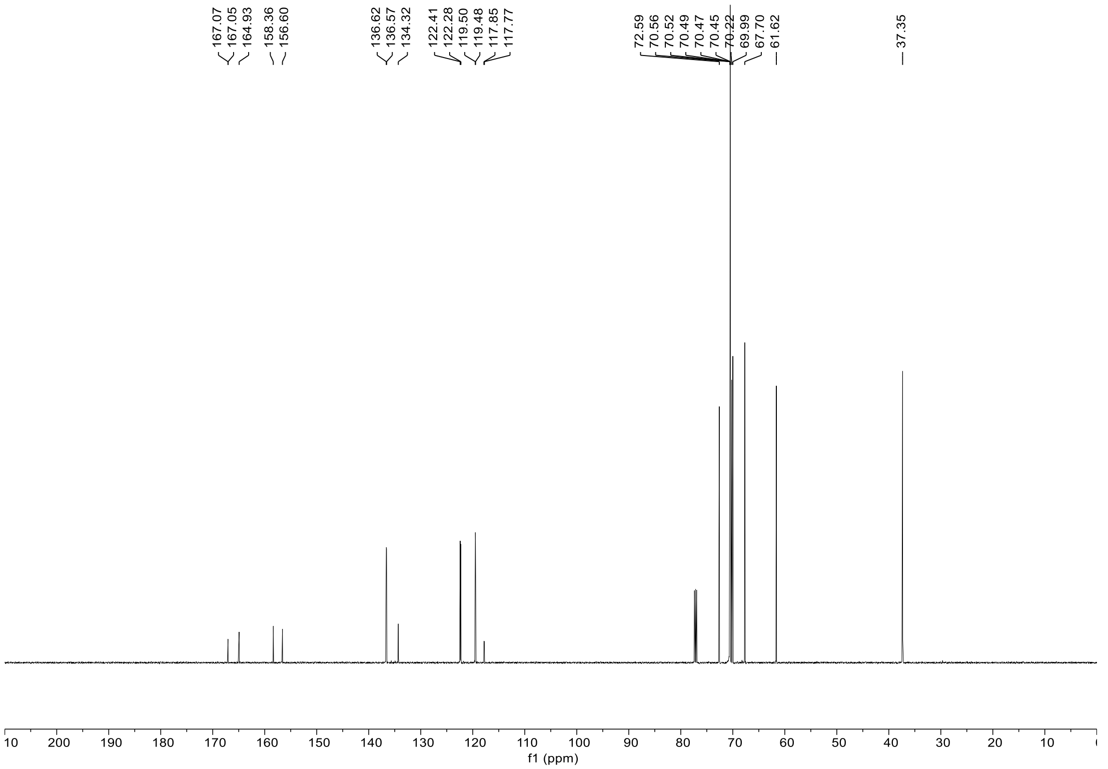

^{13}C NMR spectrum of **6** (150 MHz, $CDCl_3$)

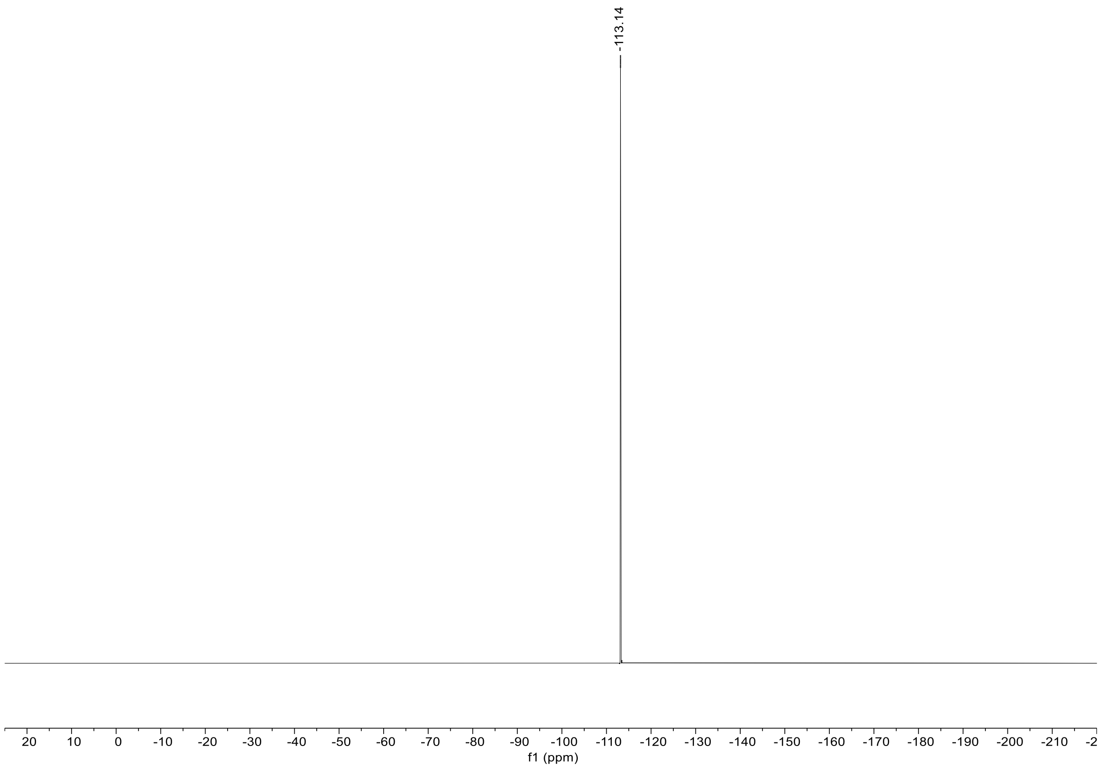

^{19}F NMR spectrum of **6** (565 MHz, $CDCl_3$)

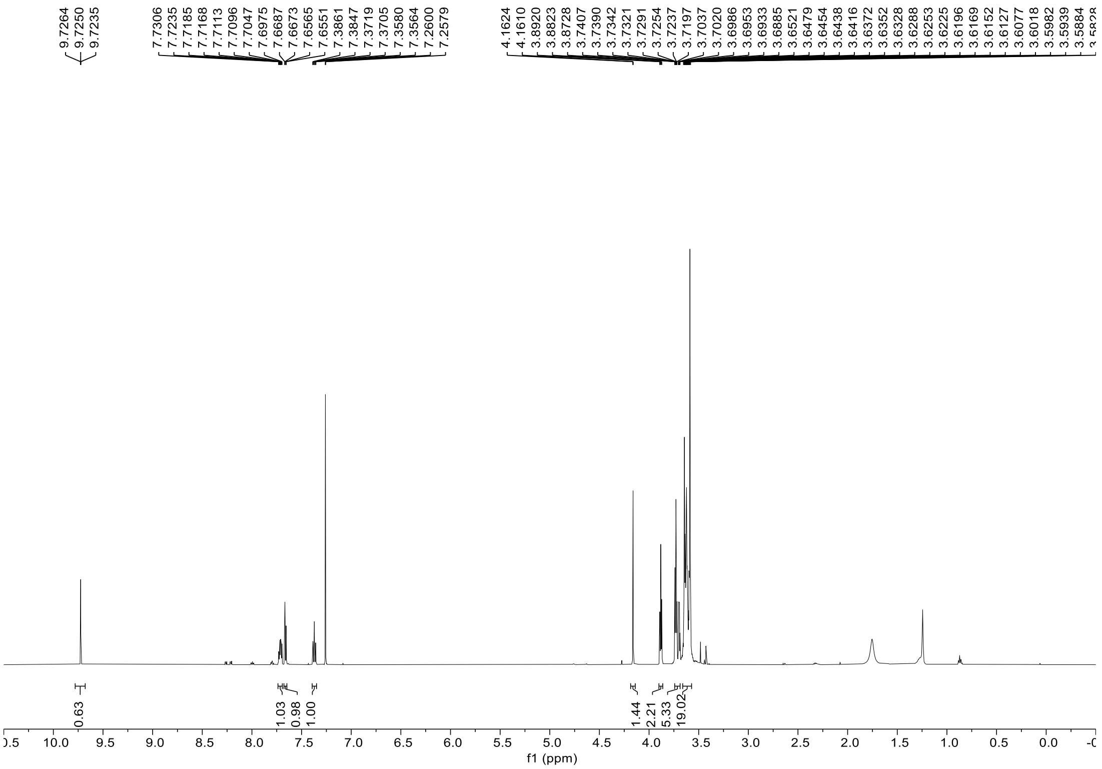

^{1}H NMR spectrum of **7** (600 MHz, $CDCl_3$)

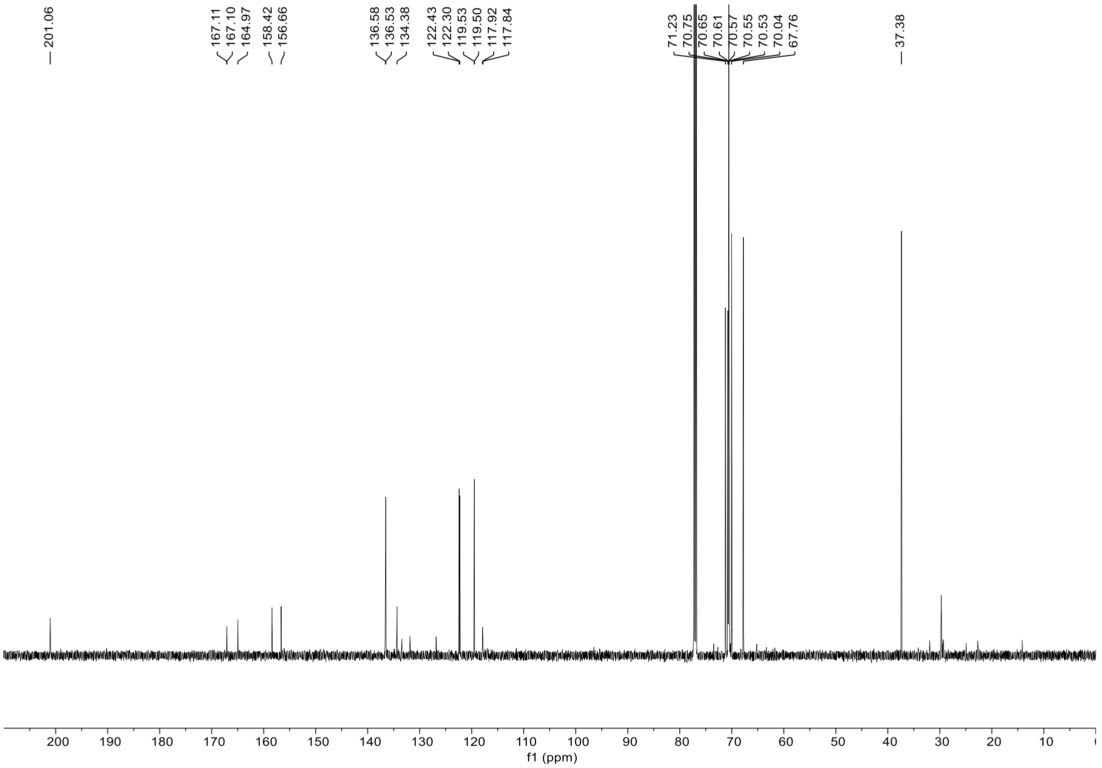

^{13}C NMR spectrum of **7** (150 MHz, $CDCl_3$)

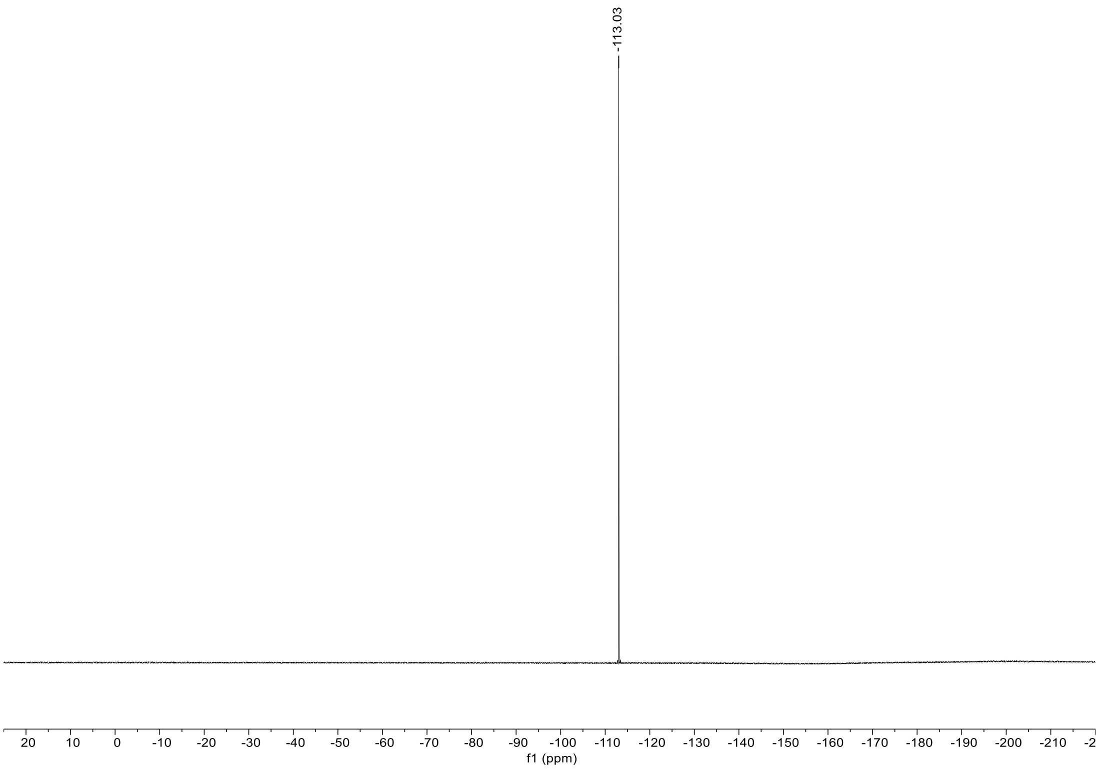

^{19}F NMR spectrum of **7** (376 MHz, $CDCl_3$)

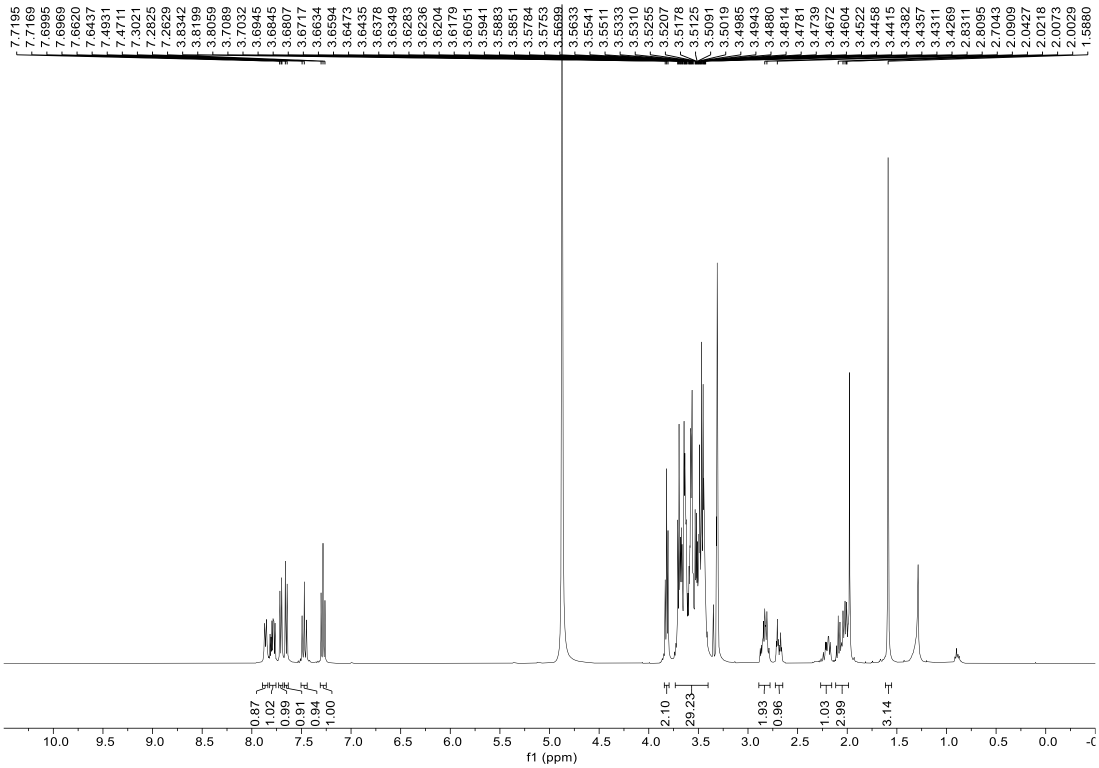

^{1}H NMR spectrum of **5** (400 MHz, CD_3OD)

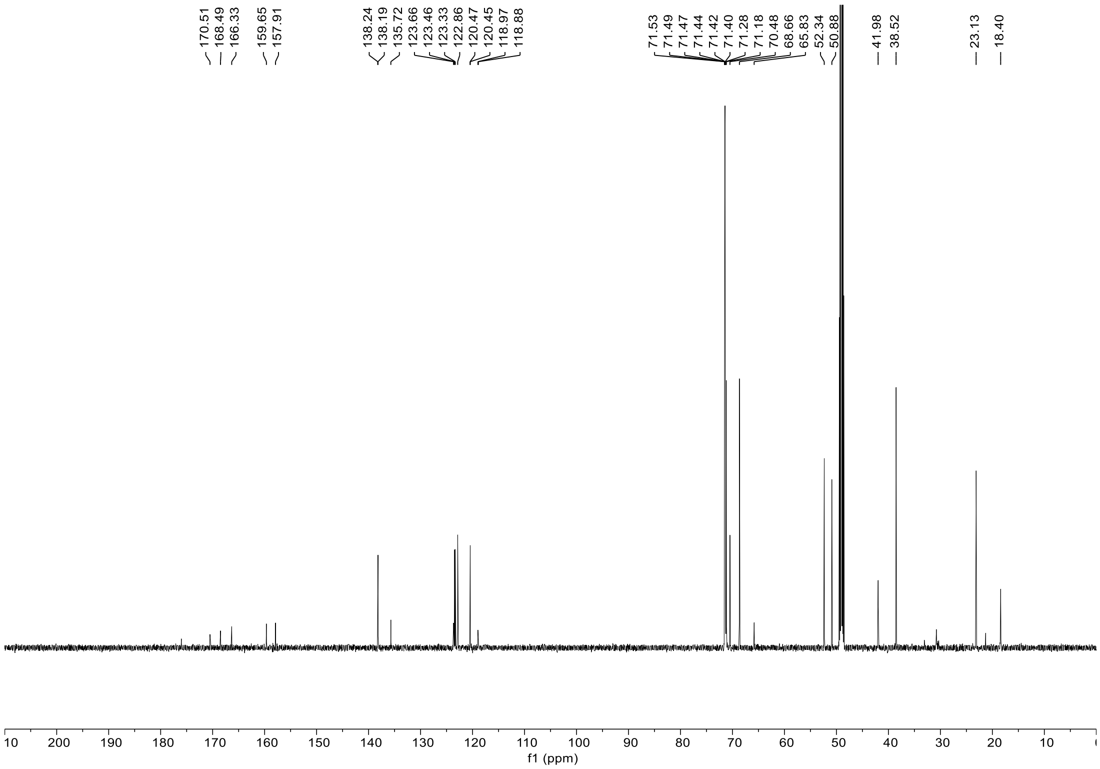

^{13}C NMR spectrum of **5** (150 MHz, CD_3OD)

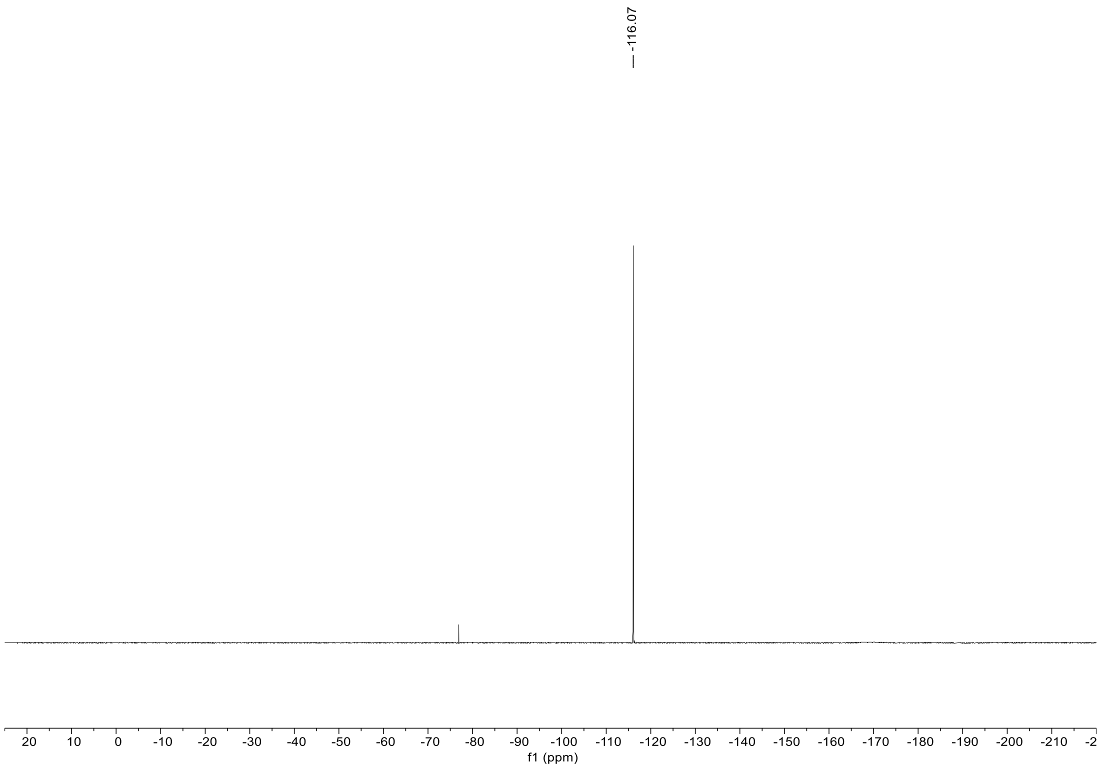

^{19}F NMR spectrum of **5** (565 MHz, CD_3OD)

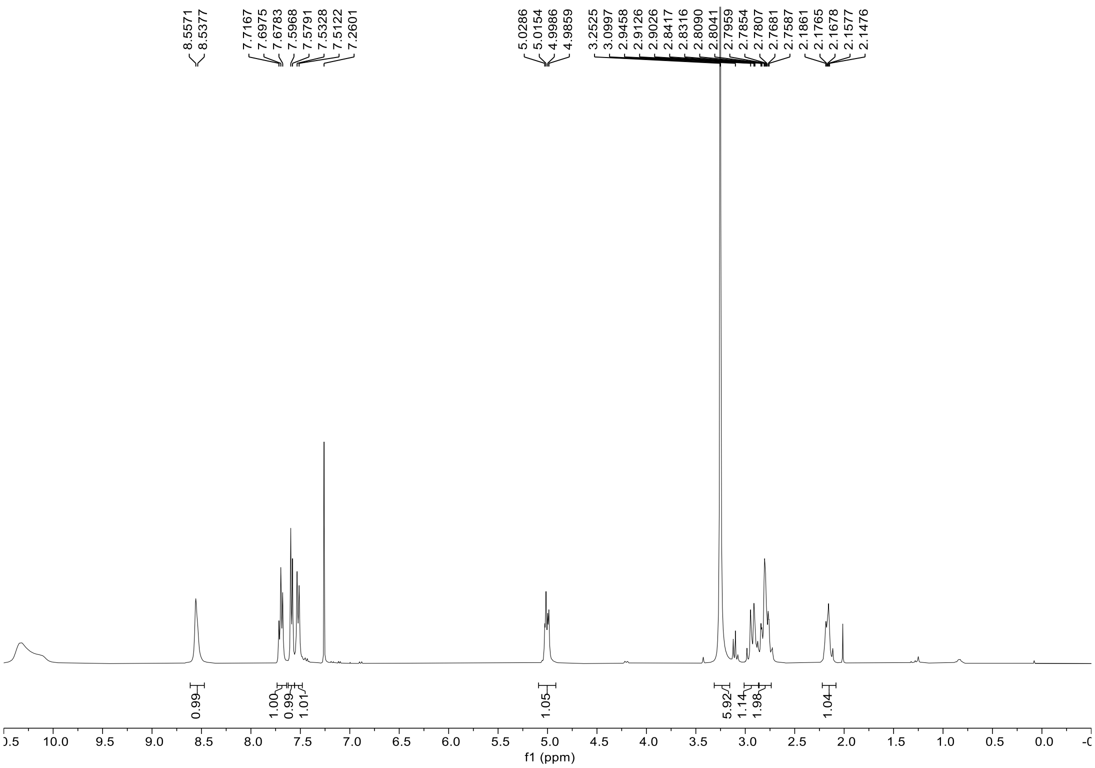

^{1}H NMR spectrum of **S1** (400 MHz, $CDCl_3$)

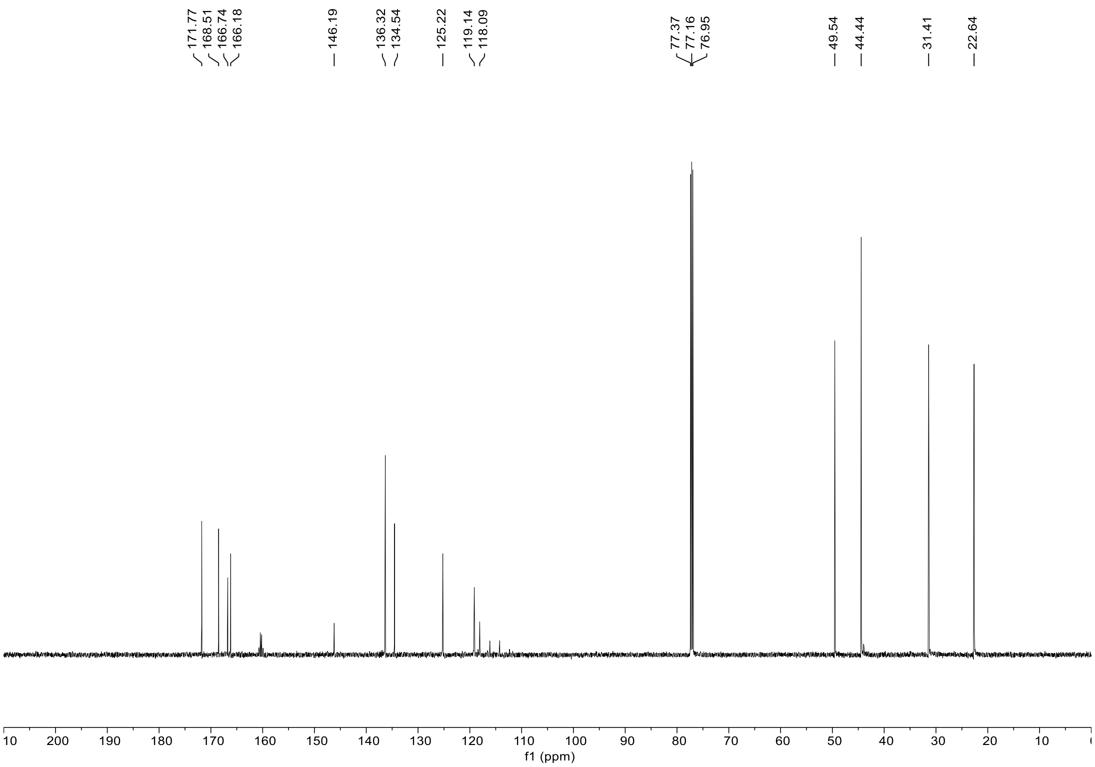

^{13}C NMR spectrum of **S1** (150 MHz, $CDCl_3$)

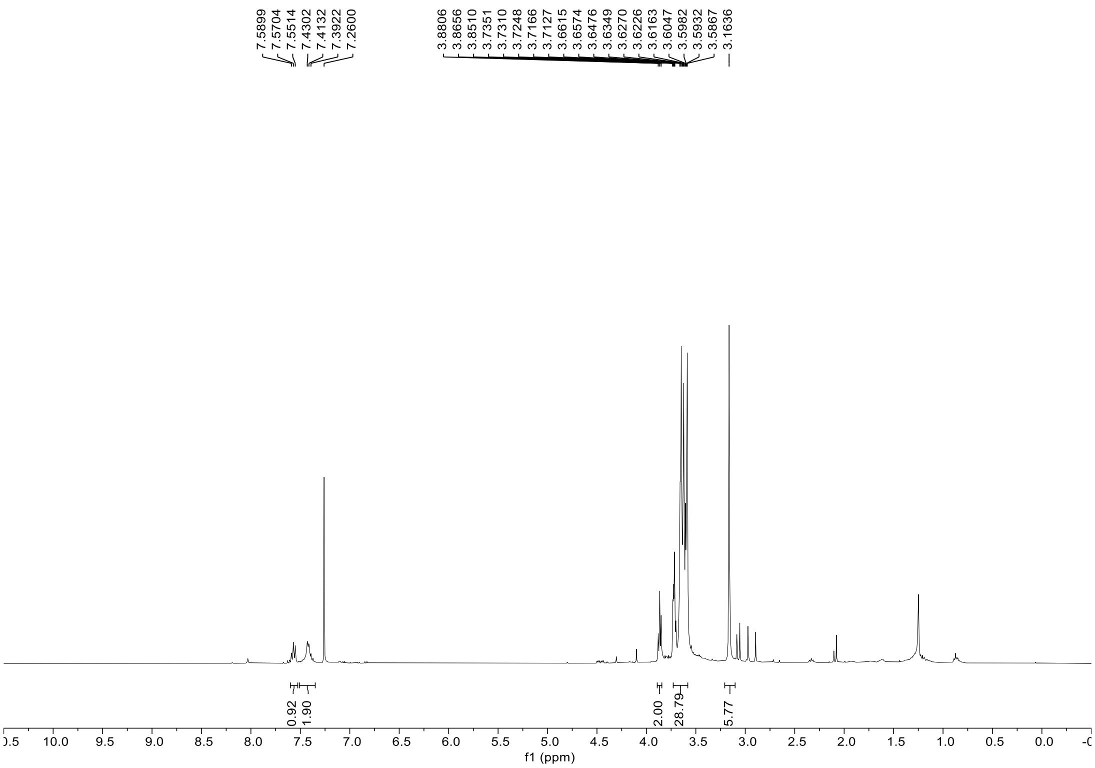

^{1}H NMR spectrum of **S2** (400 MHz, $CDCl_3$)

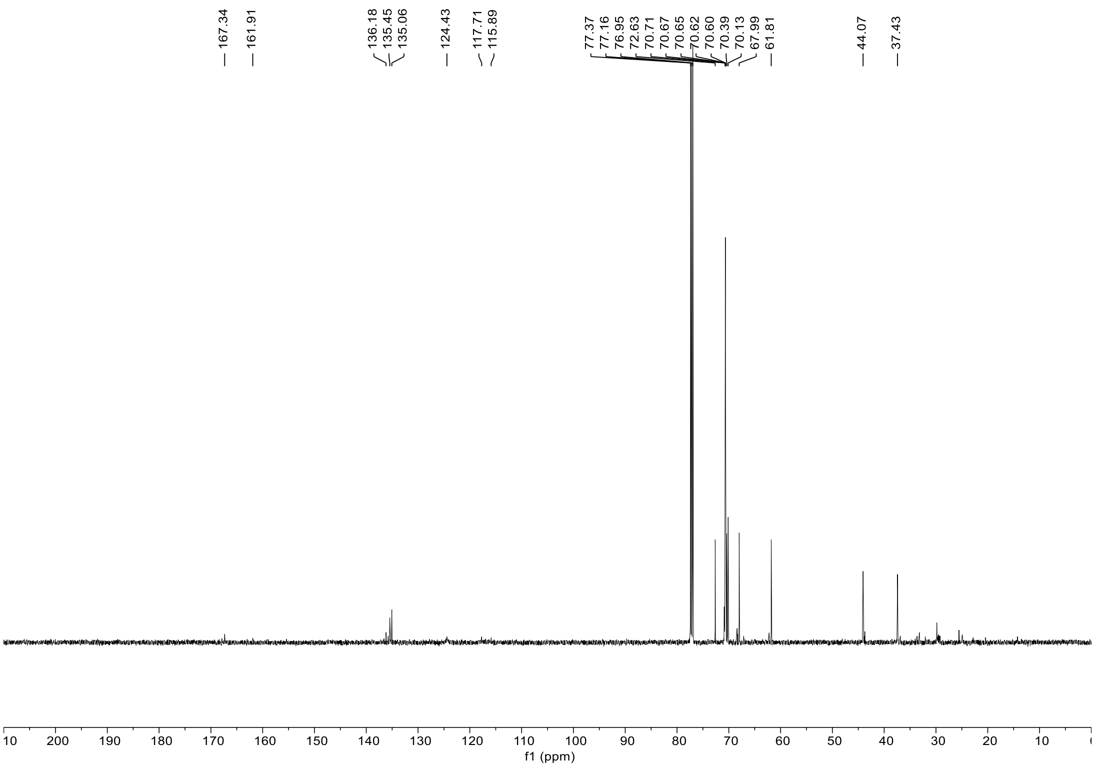

^{13}C NMR spectrum of **S2** (150 MHz, $CDCl_3$)
